# Exploring the pharmacological mechanisms of resibufogenin in castration-resistant prostate cancer via network pharmacology and experimental validation

**DOI:** 10.3389/fonc.2026.1799626

**Published:** 2026-04-14

**Authors:** Yuanxin Xing, Lu Xiang, Yanfei Jia, Yong Yang

**Affiliations:** 1 Experimental Center, Shandong University of Traditional Chinese Medicine, Jinan, China; 2 Research Center of Basic Medicine, Jinan Central Hospital, Shandong First Medical University, Jinan, China; 3 First Clinical Medical College, Shandong University of Traditional Chinese Medicine, Jinan, China

**Keywords:** BRAF, castration-resistant prostate cancer, network pharmacology, reactive oxygen species, resibufogenin

## Abstract

**Background:**

Resibufogenin (RBG) has shown anti-tumor effects in many cancers, but its role and mechanism in castration-resistant prostate cancer (CRPC) are still unclear.

**Methods:**

We screened a library of reactive oxygen species (ROS)-related compounds and selected RBG for further study. We evaluated its anti-proliferative effects in PC3 and RM1 cells by CCK-8 assay, colony formation assay, ROS detection, and Western blot. We explored the possible targets and pathways of RBG in PCa by network pharmacology and clinical data analysis. We then studied the key candidate targets by molecular docking and molecular dynamics (MD) simulation.

**Results:**

Among 20 ROS-related compounds, RBG showed the strongest inhibitory effect on PC3 cell viability. *In vitro* experiments showed that RBG inhibited the proliferation and colony formation of PC3 and RM1 cells in a dose-dependent and time-dependent manner. RBG also increased intracellular ROS levels and changed the expression of apoptosis-related proteins. Network pharmacology and KEGG analysis showed that the MAPK signaling pathway may play an important role in the action of RBG in prostate cancer. Clinical data analysis further narrowed the possible targets to BRAF and SRC. Molecular docking showed that RBG had stronger predicted binding to BRAF than to SRC. MD simulation supported the structural stability of the BRAF-RBG complex, and free energy landscape analysis further suggested the presence of a relatively stable conformational state during the simulation.

**Conclusions:**

This study suggests that RBG inhibits malignant phenotypes of CRPC cells and may exert its anti-tumor effects in part through interaction with BRAF and modulation of the MAPK signaling pathway. These findings support RBG as a promising candidate for further study in prostate cancer treatment.

## 1 Introduction

Prostate cancer (PCa) remains a significant global health burden, ranking as the second most common malignancy among men (1). Localized PCa can often be treated with surgery or radiotherapy. But once the disease progresses to castration-resistant prostate cancer (CRPC), treatment becomes much harder because the tumor no longer responds well to androgen deprivation therapy (ADT) and other systemic treatments (2–4). CRPC is a lethal stage of prostate cancer and there are still not enough effective treatment options. Prostate cancer is mainly driven by androgen receptor (AR) signaling. But other pathways also take part in tumor progression and treatment resistance, such as PI3K-AKT signaling, PTEN loss-related changes, and lineage plasticity-associated programs (5). Because of this, new small molecules with new mechanisms are still needed, especially for advanced disease such as CRPC.

Reactive oxygen species (ROS) are important in cancer. Abnormal ROS production and redox imbalance are common in cancer cells (6). Moderate ROS can help cell survival, while excessive ROS can cause cell death (7–12). Some natural compounds have anti-cancer effects through ROS-related processes (8, 10–13). On this basis, ROS-related compound libraries may represent a valuable resource for identifying potential anti-cancer agents. Accordingly, in the present study, we used a ROS-related compound library for initial screening, although the downstream mechanism of RBG may not be exclusively ROS-dependent.

Resibufogenin (RBG) is a major bioactive bufadienolide from Chan Su (14). It has shown anti-tumor activity in several cancers. Previous studies showed that RBG can induce necroptosis, ferroptosis, and cell-cycle arrest. It can also inhibit PI3K/AKT-related signaling in different tumor types (15–24), thereby blocking downstream activation of GSK3β and mTOR. In some cancer models, it can also suppress angiogenesis, migration, and invasio (23, 25). These studies show that RBG has broad anti-tumor potential. They also show that its mechanism may vary among tumor types. But its effect in CRPC and its possible target context in prostate cancer are still unclear.

**Figure 1:**
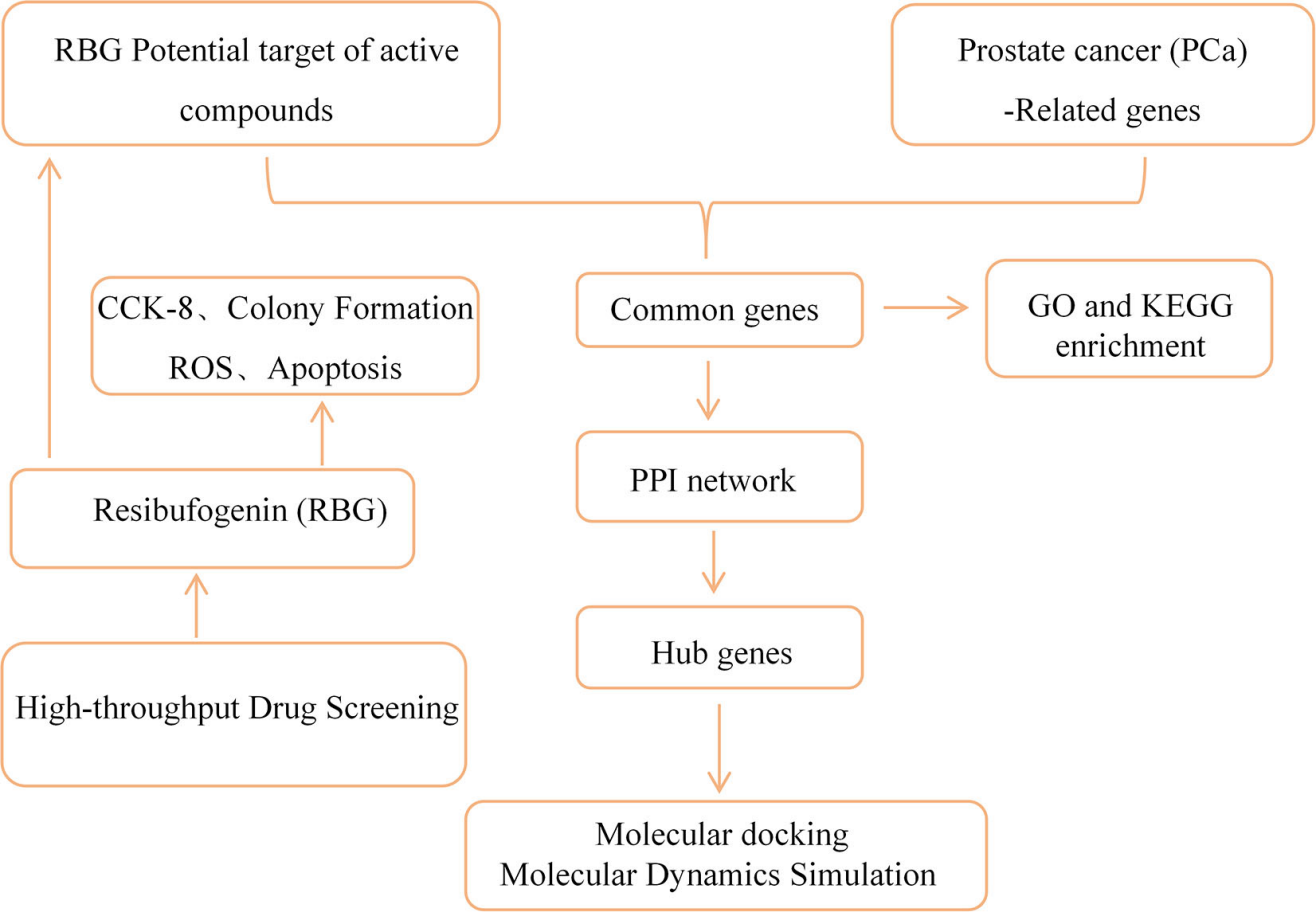
Diagram showing the study workflow.

In this study, we first screened 20 compounds from a ROS-related compound library and identified active compounds in CRPC-related cell models. After selecting RBG, we tested its effects in PC3 and RM1 cells. We then used network pharmacology and clinical data analysis to narrow a broad list of predicted targets to a smaller group of clinically relevant candidate genes. We next used molecular docking and molecular dynamics (MD) simulations to study the possible interaction between RBG and selected targets at the structural level. We also measured intracellular ROS levels after RBG treatment to support the screening background. But ROS is not presented here as the only mechanism of RBG activity. Overall, this study provides an initial biological and computational framework for understanding how RBG may act in CRPC. It also offers testable ideas for future mechanistic studies. An overview of the study design and analysis workflow is shown in **Figure 1**.

## 2 Materials and methods

### 2.1 Cell culture

PC3 and RM1 cells were cultured in RPMI-1640 medium with 10% FBS at 37°C with 5% CO_2_.

### 2.2 CCK-8 assay

Cells were seeded into 96-well plates and then treated with different concentrations of RBG for 24, 48, or 72 h. CCK-8 reagent was added to each well and incubated for 1 h. Absorbance was measured at 450 nm. Cell viability was then calculated.

### 2.3 Colony formation assay

Cells were seeded into six-well plates at a density of 800 cells per well. The cells were then treated with RBG and cultured for 14 days to allow colony formation. The colonies were fixed with paraformaldehyde, stained with crystal violet, and counted.

### 2.4 Reactive oxygen species

ROS levels were measured with a ROS Assay Kit (Beyotime) according to the manufacturer’s instructions. After treatment, cells were stained with DCFH-DA. The cells were then imaged under the same microscope settings, and fluorescence intensity was quantified with ImageJ.

### 2.5 Western blot

Western blot was performed as previously described with minor modifications (26). Primary antibodies against Bax (Abcam, #AB32503), Bcl-2 (Proteintech, #12789-1-AP), and β-actin (Proteintech, #66009-1-Ig) were used in this study.

### 2.6 Bioinformatics analysis

Potential targets of RBG were predicted with SwissTargetPrediction (https://swisstargetprediction.ch/) and PharmMapper (https://www.lilab-ecust.cn/pharmmapper/). PCa-related targets were collected from GeneCards (https://www.genecards.org/). Shared targets were identified with a Venn diagram.

The shared targets were imported into STRING (https://cn.string-db.org/) to build a protein–protein interaction (PPI) network. The network was analyzed with Cytoscape (v3.9.1). The MCODE and CytoHubba plugins were used to calculate Degree, Betweenness, and Closeness values (27). Based on these results, 31 hub genes were identified.

The shared targets were further analyzed with DAVID (https://davidbioinformatics.nih.gov/) for Gene Ontology (GO) and Kyoto Encyclopedia of Genes and Genomes (KEGG) enrichment. Survival analysis was performed with GEPIA2 (http://gepia2.cancer-pku.cn/#index).

### 2.7 Molecular docking and molecular dynamics

The crystal structures of the selected target proteins were obtained from the RCSB Protein Data Bank (PDB). Molecular docking was performed with AutoDock Vina. The pose with the lowest binding energy was selected for further analysis (28).

Molecular dynamics (MD) simulation was performed with GROMACS to test the stability of the protein–ligand complex. The protein topology was generated with the CHARMM36 all-atom force field. The ligand topology was generated with Sobtop and mainly assigned with the general AMBER force field (GAFF). The complex was placed in a cubic box. It was then solvated with the TIP3P water model and neutralized with counterions at a final salt concentration of 0.15 M.

Energy minimization was performed with the steepest descent algorithm. NPT equilibration was then carried out. After that, a 100 ns production run was performed. The trajectory was analyzed for RMSD, RMSF, Rg, hydrogen bonds, SASA, and free energy landscape. Before RMSD analysis, the trajectory was recentered and compacted to reduce periodic boundary artifacts. Backbone fitting was then applied. The free energy landscape was generated with the gmx sham module in GROMACS (29).

### 2.8 Statistical analysis

All quantitative data were collected from at least three independent experiments. Results are presented as mean ± SD. Statistical analyses and graph generation were carried out using GraphPad Prism. Comparisons between two groups were performed using a two-tailed Student’s t-test. Comparisons among multiple groups were performed using one-way ANOVA followed by appropriate *post hoc* tests. A value of P < 0.05 was considered statistically significant.

## 3 Results

### 3.1 Screening identified resibufogenin as a potential PCa inhibitor

**Figure 2:**
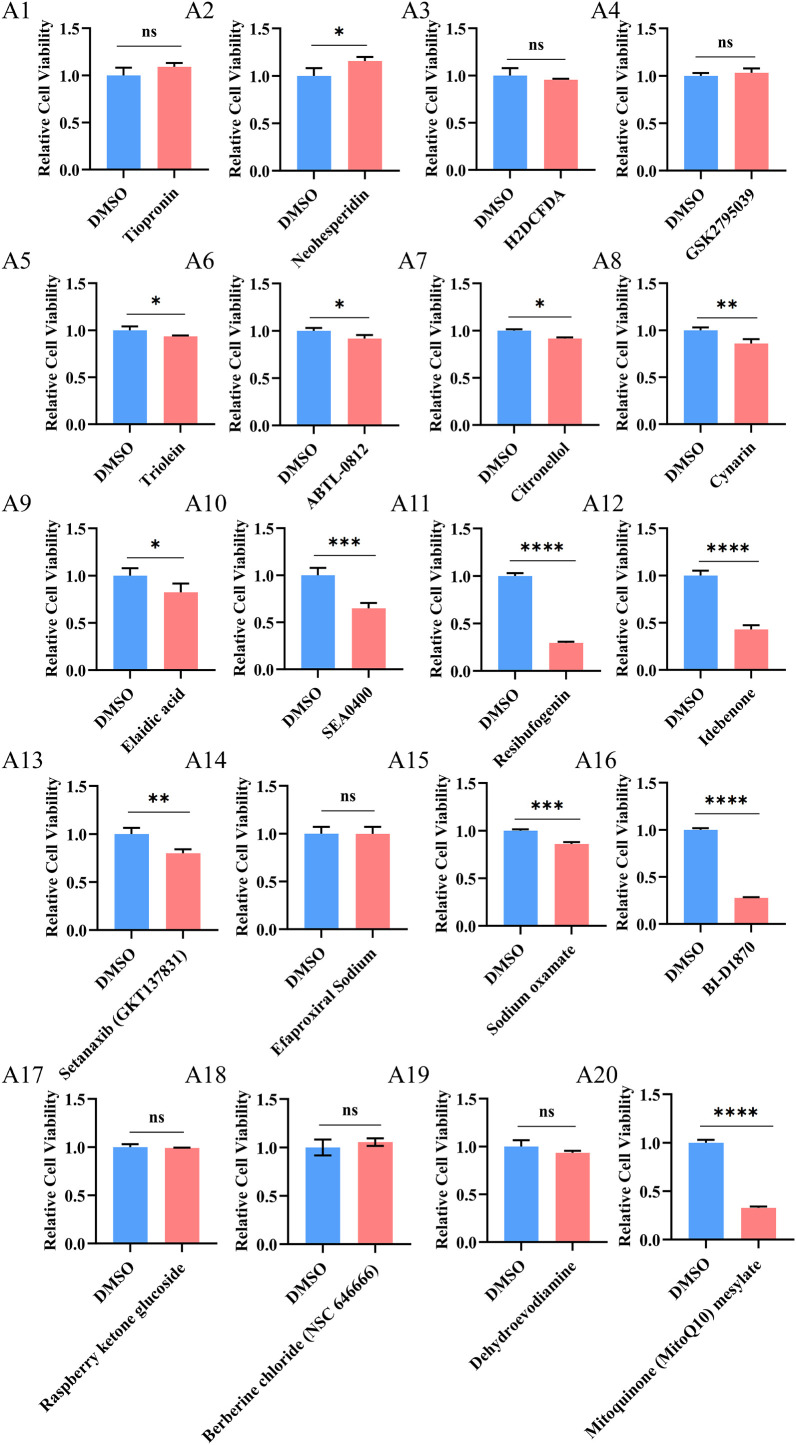
RBG as a potent inhibitor of PCa cell viability. (A1-A20) PC3 cells were treated with 20 ROS-related compounds, and cell viability was assessed. *: p<0.05, **: p<0.01, ***: p<0.001, ****: p<0.0001, ns: not significant (p≥0.05).

We selected 20 ROS-related compounds from the Selleck compound library and tested their cytotoxicity in PC3 cells. Some compounds showed moderate inhibitory effects. RBG showed the strongest effect and reduced cell viability by more than 70% at the screening concentration (**Figure 2**). Based on this result, we selected RBG for further study.

### 3.2 RBG suppresses cell proliferation and induces oxidative stress in AR-insensitive prostate cancer models

**Figure 3:**
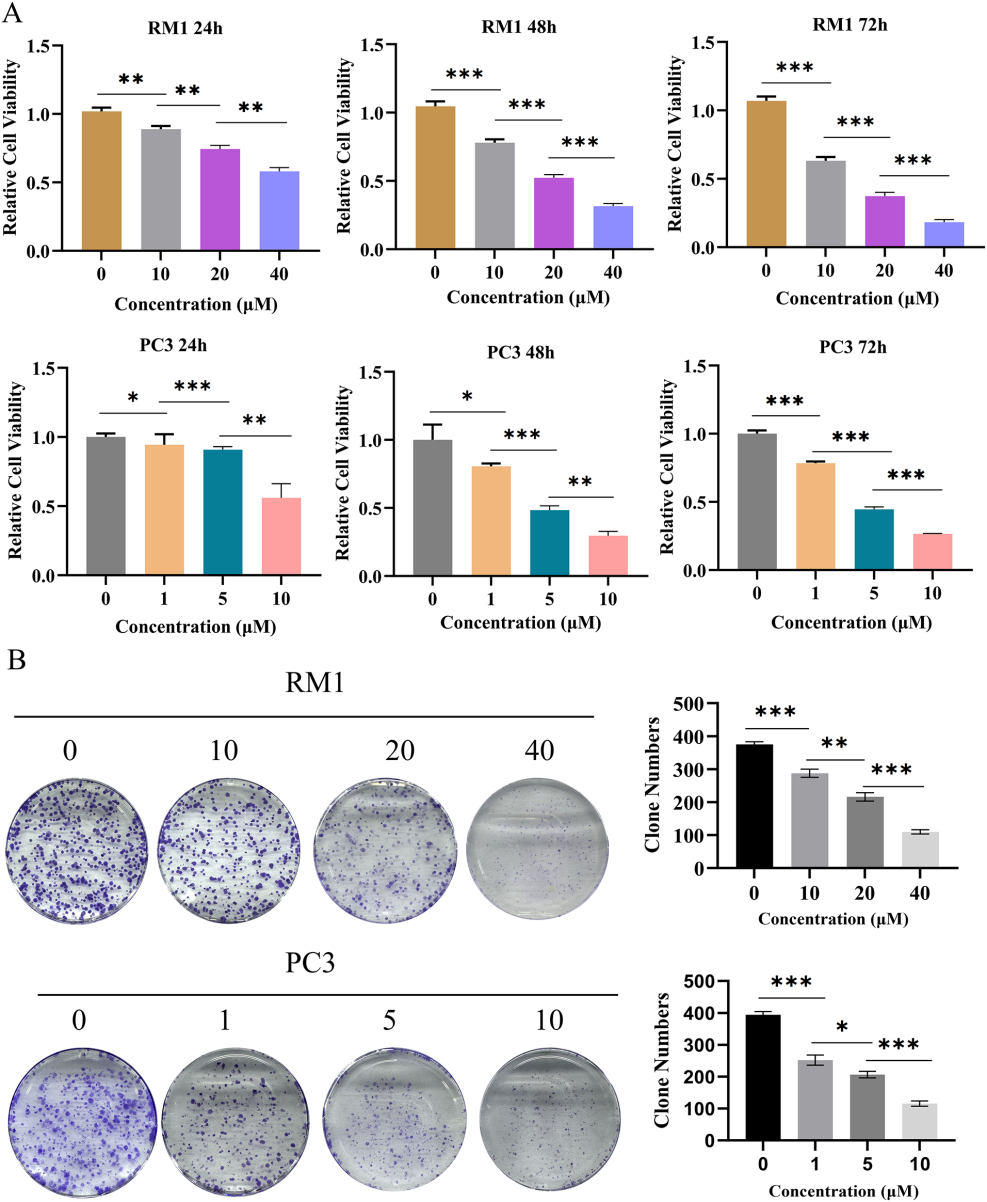
RBG inhibits proliferation and clonogenicity of PCa cells. **(A)** RBG treatment reduced the viability of RM1 and PC3 cells in a concentration- and time-dependent manner, as determined by CCK-8 assays at 24h, 48h, and 72 h. **(B)** Colony formation assays showed that RBG suppressed the clonogenic capacity of RM1 and PC3 cells in a concentration- and time-dependent fashion. *: p<0.05, **: p<0.01, ***: p<0.001.

To confirm the screening result, we treated RM1 and PC3 cells with different concentrations of RBG. CCK-8 assays showed that RBG inhibited cell proliferation in both cell lines in a dose-dependent and time-dependent manner (**Figure 3A**). Colony formation assays showed similar results. RBG also reduced the colony-forming ability of both RM1 and PC3 cells after treatment (**Figure 3B**). These findings show that RBG clearly inhibited the growth of CRPC cells.

**Figure 4:**
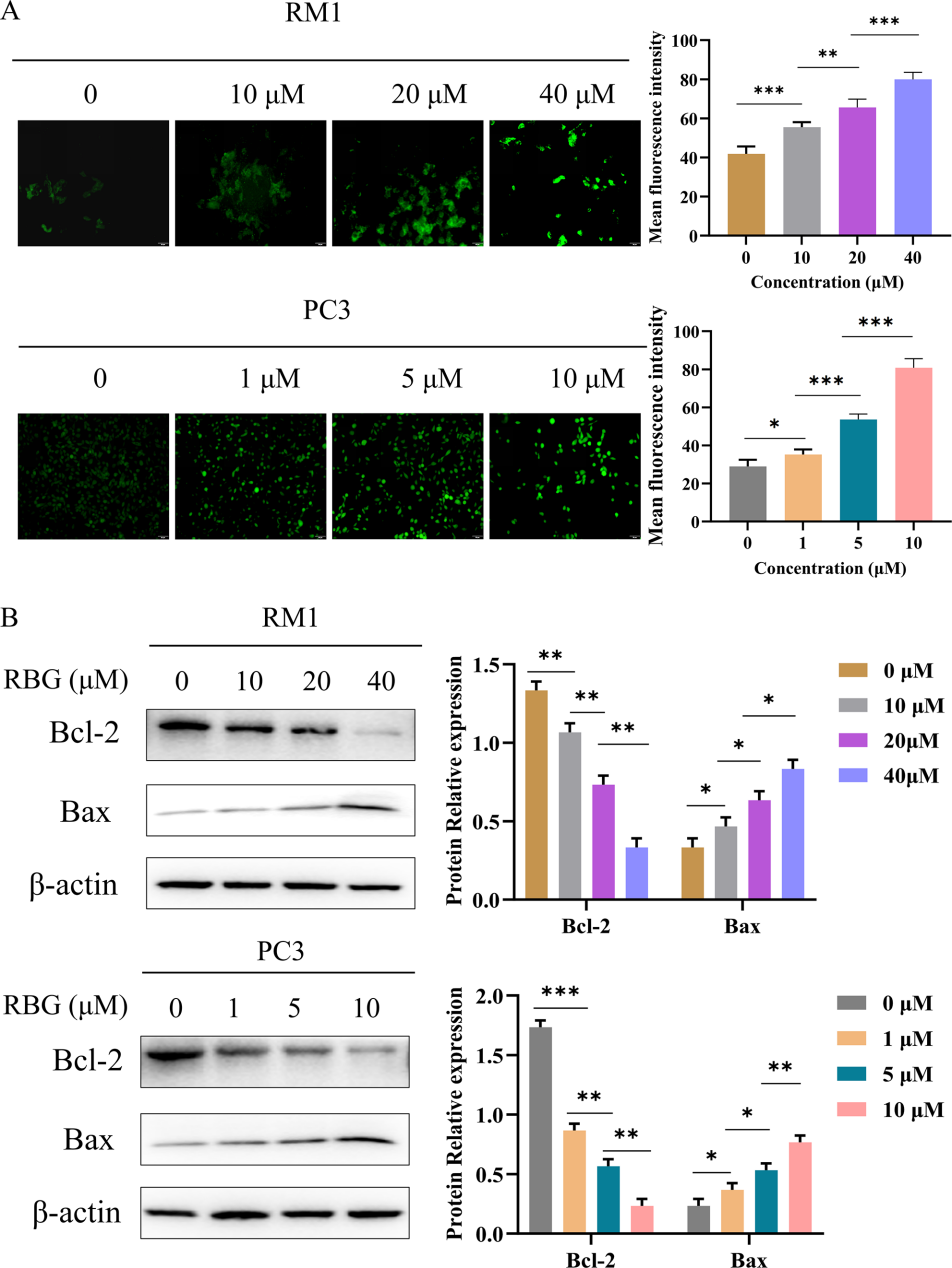
RBG elevates intracellular ROS and modulates apoptosis-associated markers in RM1 and PC3 cells. **(A)** Representative DCFH-DA fluorescence images and corresponding quantification of mean fluorescence intensity in RM1 and PC3 cells treated with RBG. **(B)** Western blot analysis of Bcl-2 and Bax in RM1 and PC3 cells treated with RBG. *: p<0.05, **: p<0.01, ***: p<0.001.

We then measured intracellular ROS levels after RBG treatment with the DCFH-DA fluorescent probe. RBG increased ROS-associated fluorescence in both cell lines. The signal also increased with dose (**Figure 4A**). This result supports the screening background based on ROS-related compounds. It also suggests that RBG can increase oxidative stress in these models.

We also examined the apoptosis-related proteins Bcl-2 and Bax. Western blot results showed that RBG decreased Bcl-2 expression and increased Bax expression in both RM1 and PC3 cells (**Figure 4B**). These data support the growth-inhibitory effect of RBG and suggest that RBG treatment is accompanied by changes in cell death-related signaling.

### 3.3 Identification of hub targets of RBG in PCa using network pharmacology

**Figure 5:**
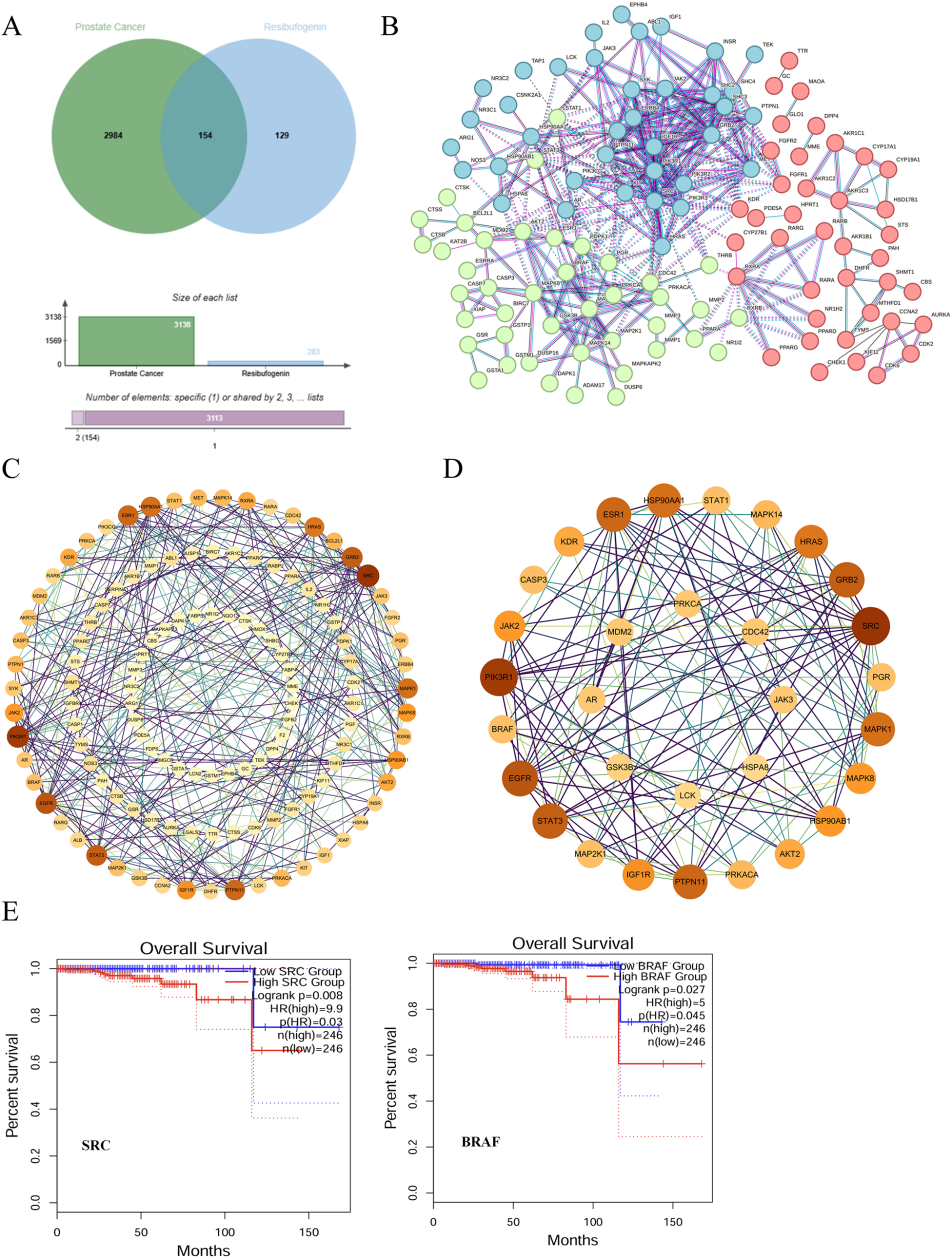
Identification of hub targets of RBG in PCa using network pharmacology. **(A)** Venn diagram showing the overlap between PCa-related genes (3183) and predicted RBG targets (283 total = 154 shared + 129 unique). **(B)** The PPI network of RBG-related targets in PCa. **(C)** Topological analysis of the PPI network. **(D)** Core hub-gene module extracted from the PPI network. **(E)** Kaplan-Meier survival analysis of BRAF and SRC.

We collected 283 putative targets of RBG from the SwissTargetPrediction and PharmMapper databases. We also collected prostate cancer-related genes (n = 3183) from GeneCards. After overlap analysis, we identified 154 shared genes between the predicted RBG targets and the prostate cancer-related genes. The other 129 predicted targets were not annotated as prostate cancer-related in GeneCards (**Figure 5A**).

We then examined the interaction pattern of the 154 shared genes. We constructed a protein-protein interaction (PPI) network by using the STRING database. After isolated nodes were removed according to the network settings, the final network contained 123 nodes and 329 edges (P < 1.0e−16) (**Figure 5B**). We next analyzed the network topology in Cytoscape (**Figures 5B, C**). Based on topological parameters and module analysis, we identified a dense cluster of 31 hub genes (**Figure 5D**). These genes included PGR, AKT2, MAPK1, and other regulatory genes. They represented the core candidate target set of RBG in prostate cancer.We then analyzed the 31 hub genes with transcriptomic and survival data from GEPIA2 to improve the clinical relevance of the network pharmacology results. We first examined their expression patterns in prostate cancer. We then evaluated their association with patient survival. Among the 31 hub genes, only BRAF and SRC met both criteria. Both genes showed significant dysregulation in prostate cancer, and higher expression was associated with poorer patient survival (**Figure 5E**; **Supplementary Figure 5**). Based on these results, we selected BRAF and SRC as candidate targets for further structural analysis.

### 3.4 GO and KEGG enrichment analysis

**Figure 6:**
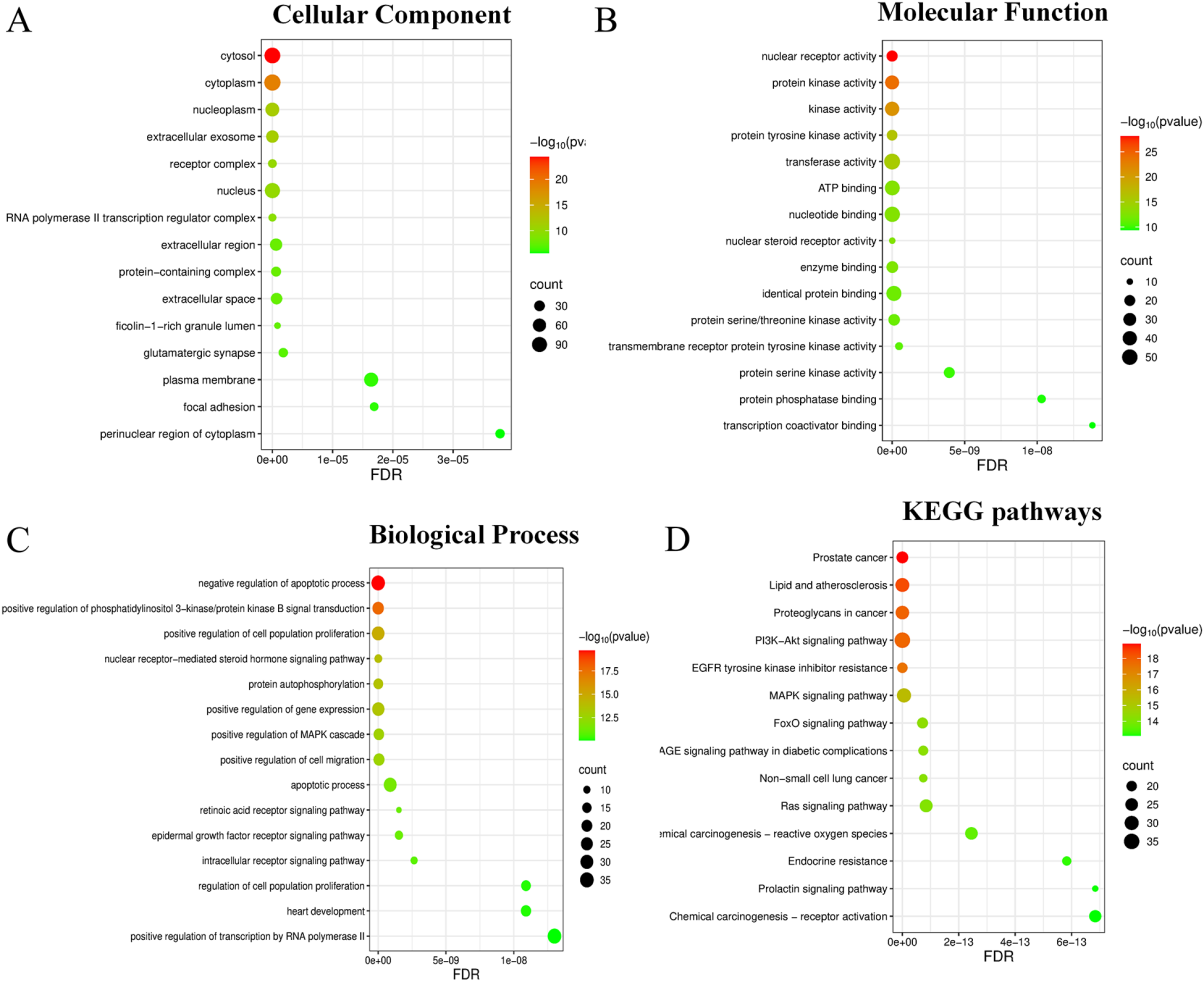
Top 15 enriched GO terms and KEGG pathways for the target genes. The results are categorized into: **(A)** Biological Process, **(B)** Cellular Component, **(C)** Molecular Function, and **(D)** KEGG pathways.

We next performed GO enrichment analysis to examine the biological features of the shared targets. The enriched terms were grouped into biological process, cellular component, and molecular function (**Figures 6A–C**).

We also performed KEGG pathway enrichment analysis to identify the pathways that may be involved in the action of RBG in prostate cancer. A total of 165 pathways met the significance threshold. The most enriched pathways included Proteoglycans in cancer, Chemical carcinogenesis - reactive oxygen species, the PI3K-AKT signaling pathway, and the MAPK signaling pathway (**Figure 6D**). These results suggest that RBG may affect several cancer-related signaling pathways, not just one.

### 3.5 Molecular docking and molecular dynamics analysis supported a stable interaction between RBG and BRAF

**Figure 7:**
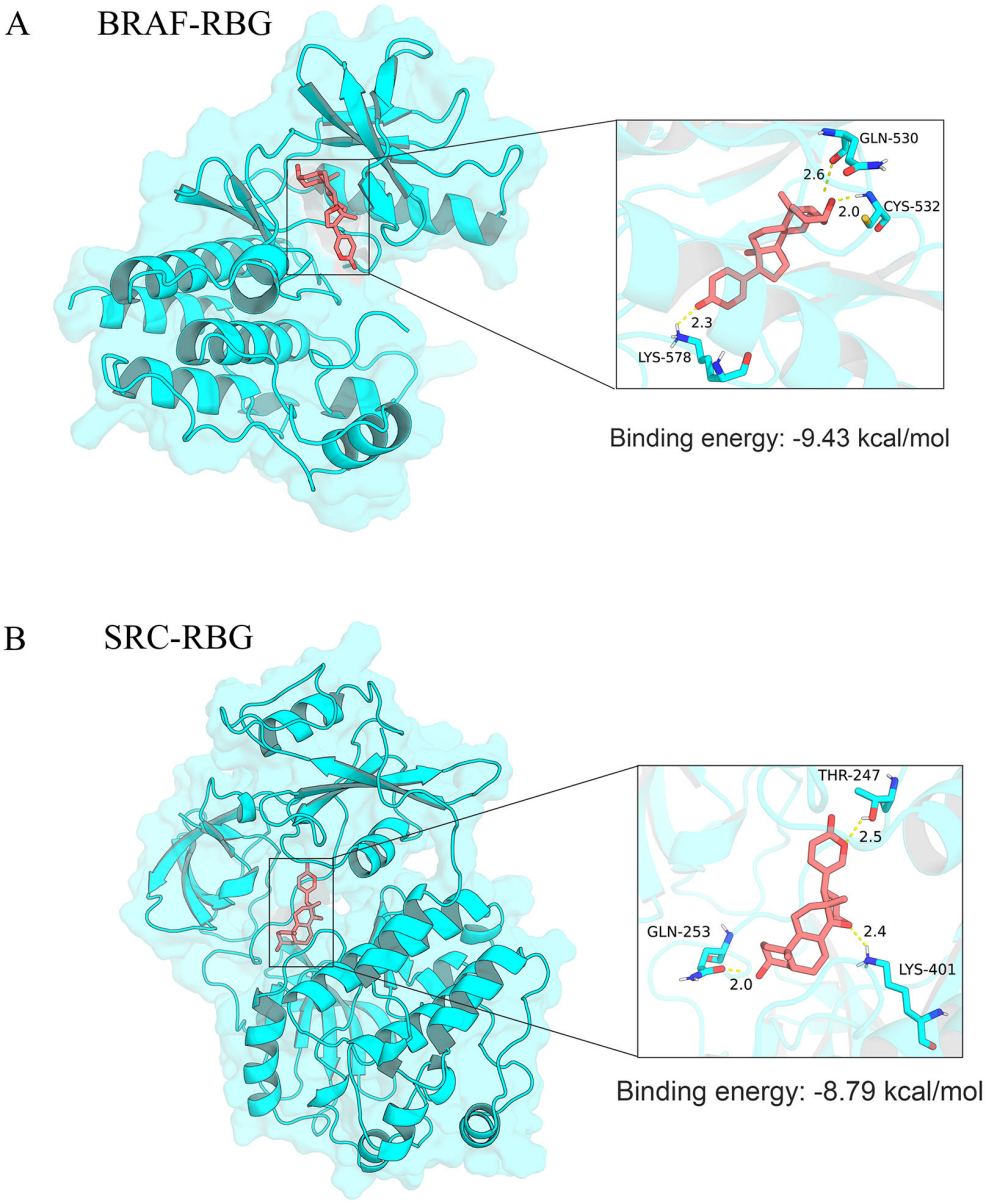
Binding mode visualization of RBG to target proteins. The diagram is generated based on molecular docking results, depicting the predicted binding patterns of RBG with **(A)** BRAF and **(B)** SRC. For each panel, the upper section presents an overall view of the binding site, while the lower section provides a detailed close-up.

Based on the survival analysis results, we obtained the crystal structures of BRAF and SRC from the Protein Data Bank (PDB). All selected structures had a resolution below 3.0 Å and were suitable for docking analysis. Molecular docking showed that RBG had stronger predicted binding to BRAF (-9.43 kcal/mol) than to SRC (-8.79 kcal/mol). In the docking model, RBG occupied the kinase-binding pocket of BRAF and formed hydrogen-bond and hydrophobic interactions with residues in the binding region. This suggested a favorable binding mode (**Figure 7A**). In contrast, the predicted interaction between RBG and SRC was weaker and showed less favorable binding characteristics (**Figure 7B**).

AR signaling is the main driver of prostate cancer. But bypass pathways may also contribute to CRPC progression. BRAF also showed a stronger predicted interaction with RBG in the structural analysis. Based on this, we selected BRAF for further molecular dynamics analysis (30–32).

**Figure 8:**
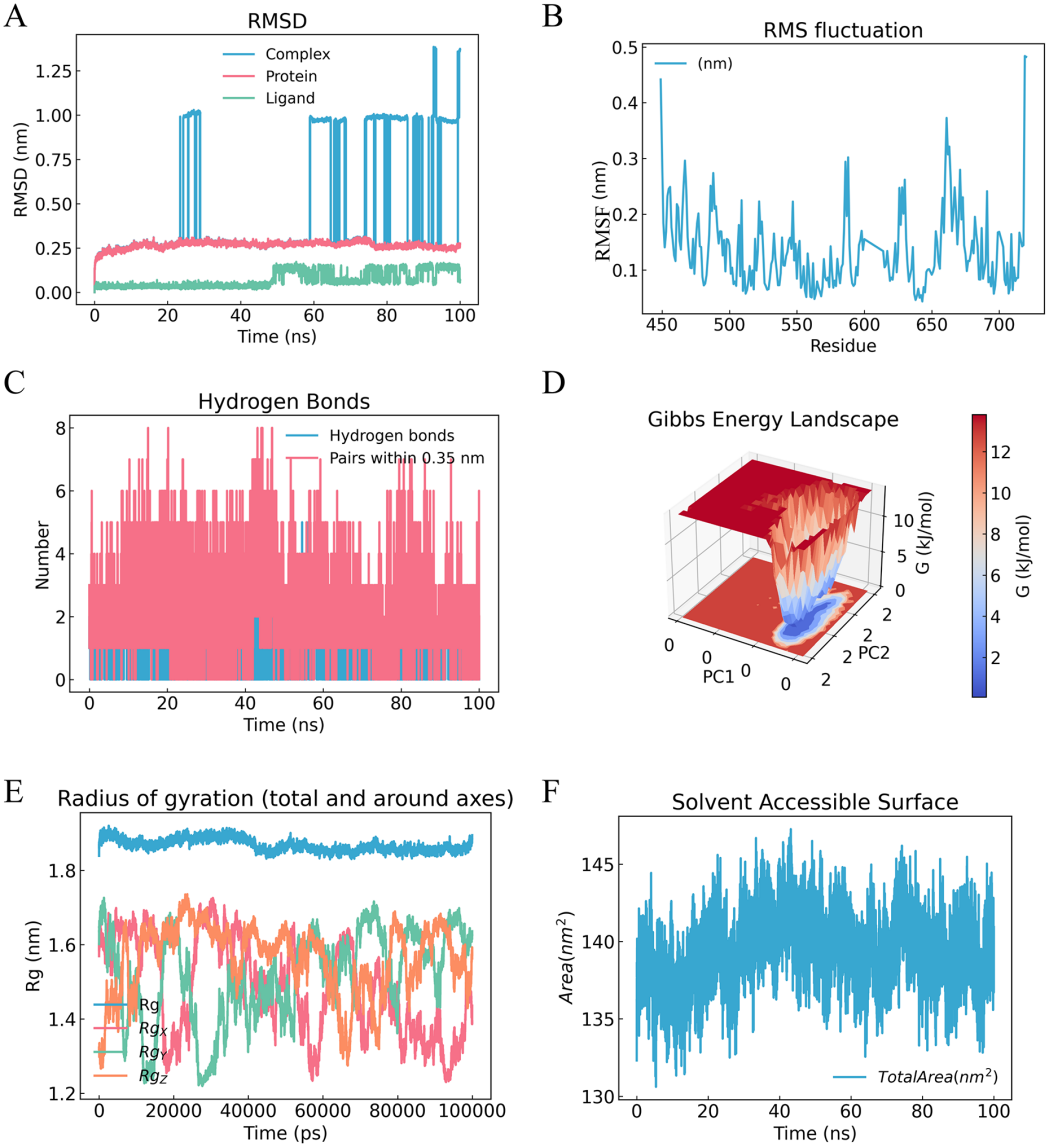
MD simulation analysis of the BRAF-RBG complex. **(A)** RMSD over time. **(B)** RMSF by residue. **(C)** Changes in the number of hydrogen bonds between the ligand and receptor during the simulation. **(D)** Free energy landscape of the BRAF-RBG complex based on the MD trajectory. **(E)** Rg over time. **(F)** SASA over time.

We performed a 100 ns molecular dynamics simulation for the BRAF-RBG complex. The RMSD curve showed that the complex became relatively stable after about 20 ns and stayed stable during the rest of the simulation (**Figure 8A**). RMSF analysis showed lower fluctuations in several residues around the binding pocket (**Figure 8B**). The number of hydrogen bonds between RBG and BRAF stayed within a relatively stable range during the simulation, which supported sustained ligand binding (**Figure 8C**). Free energy landscape analysis based on the MD trajectory also showed a relatively stable conformational state for the BRAF-RBG complex during the simulation (**Figure 8D**). The Rg and SASA results showed no clear change in overall protein compactness or solvent exposure after ligand binding (**Figures 8E, F**). Together, these data suggest that BRAF may be a possible mediator of RBG action in a CRPC-relevant context. More biological validation is still needed.

## 4 Discussion

This study combined compound screening, *in vitro* assays, network pharmacology, clinical data analysis, and structural modeling to explore the anti-tumor effect of RBG in prostate cancer. Among 20 ROS-related compounds, RBG showed the strongest inhibitory effect on PC3 cells. Follow-up experiments in PC3 and RM1 cells showed that RBG inhibited cell proliferation and colony formation in a dose-dependent and time-dependent manner. RBG also increased intracellular ROS levels and changed the expression of the apoptosis-related proteins Bax and Bcl-2. These findings support a clear growth-inhibitory effect of RBG in AR-insensitive prostate cancer models.

Previous studies have linked RBG to the PI3K/AKT and Na^+^/K^+^-ATPase signaling pathways in multiple tumors (23, 24, 33), but its specific targets in PCa remain unclear. To explore the possible molecular basis of RBG action, we combined network pharmacology with clinical data to refine 31 hub genes to 2 clinically relevant candidate targets: BRAF and SRC. Notably, while our KEGG enrichment analysis suggested the PI3K-AKT pathway, structural data more clearly pointed to the MAPK/BRAF axis. This suggests that RBG may affect more than one cancer-related signaling pathway.

Molecular docking and molecular dynamics simulations supported BRAF as a possible mediator of RBG action. BRAF is a serine/threonine protein kinase encoded by the BRAF gene (34). It is a key signaling molecule in the MAPK/ERK pathway (35). It is activated by upstream signals and then phosphorylates and activates downstream MEKs (36).This process amplifies growth signals and sends them to the nucleus. BRAF is an important therapeutic target for anticancer drugs (37, 38). Recent large-scale genomic studies show that activating BRAF events are present in a small subset of prostate cancers, about 3.3%. These changes are mainly class II mutations or gene rearrangements, not the classic V600E hotspot that is common in melanoma and colorectal cancer. This suggests that BRAF-driven MAPK activation may have distinct features in prostate cancer and supports further study of MAPK-targeted strategies in this setting (32).Because BRAF activation in prostate cancer is different from that in other cancers, we selected BRAF for further analysis to examine its possible role in the response to RBG. The RBG-BRAF complex showed a favorable docking pattern and stable behavior during MD simulation. The predicted interaction between RBG and SRC was weaker. This further supported the choice of BRAF for follow-up analysis in this study. These findings suggest that RBG may be related to both redox-related responses and kinase-associated signaling in this model.

This study has some limitations. The current results are mainly based on *in vitro* models and computational prediction. So *in vivo* studies are still needed to test the overall effect of RBG. Still, RBG showed growth-inhibitory effects, increased intracellular ROS levels, and showed a predicted interaction with BRAF in structural analysis. These findings suggest that RBG may be a promising lead compound for prostate cancer treatment. This study also provides an initial framework for understanding how RBG may act in prostate cancer and shows the value of combining phenotypic screening with structural biology in drug discovery.

## 5 Conclusion

This study used experimental screening and computational analysis to examine the anti-prostate cancer potential of RBG. RBG inhibited the growth of PC3 and RM1 cells, increased intracellular ROS levels, and altered the expression of apoptosis-related proteins. Network pharmacology, clinical data analysis, molecular docking, and molecular dynamics simulation suggested that BRAF may be a candidate mediator of RBG action in a CRPC-relevant context. These findings support RBG as a promising lead compound for further study in prostate cancer.

## Data Availability

The original contributions presented in the study are included in the article/**Supplementary Material**. Further inquiries can be directed to the corresponding authors.
