# Supplementary figures and images for: Exploring the pharmacological mechanisms of resibufogenin in castration-resistant prostate cancer via network pharmacology and experimental validation
Source: Front Oncol. 2026 Apr 14;16:1799626. doi: 10.3389/fonc.2026.1799626 (PMC13120906; doi:10.3389/fonc.2026.1799626)

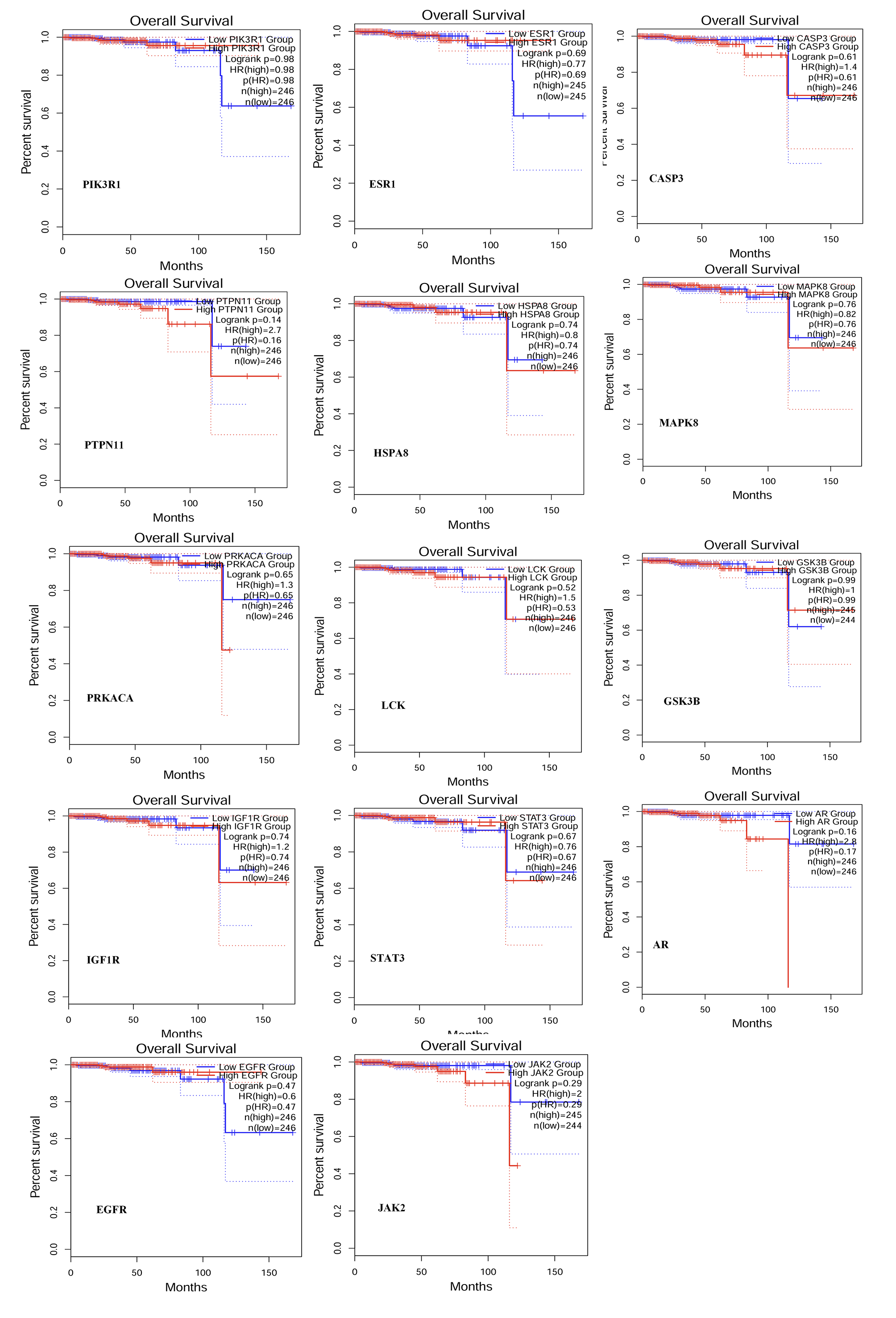

Supplement: Supplementary file 1 [file Image1.tif]

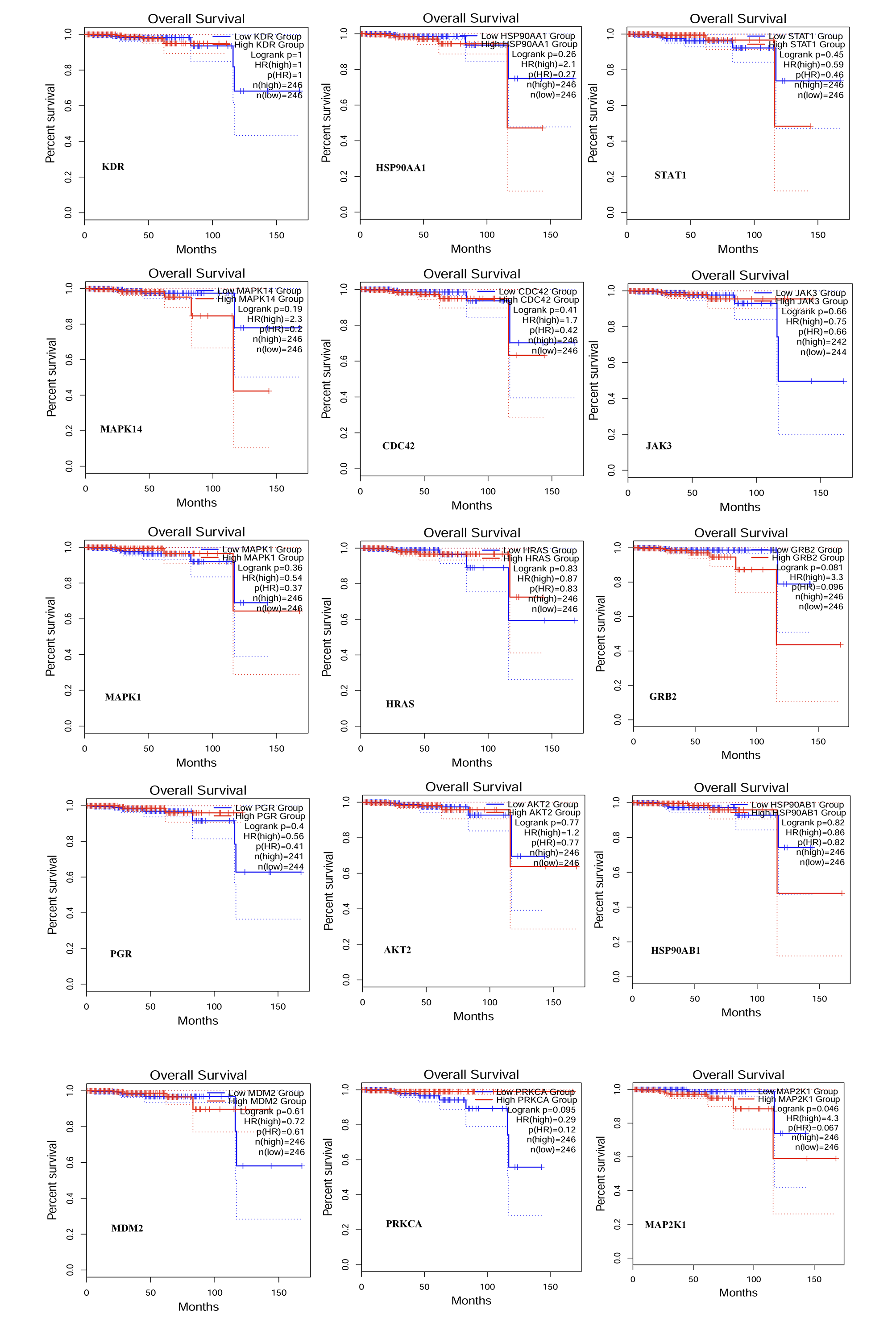

Supplement: Supplementary file 2 [file Image2.tif]
